# Society Meetings

**Published:** 1895-04

**Authors:** 


### Society Meetings.

The Association of Military Surgeons of the United States will hold its next annual meeting at Buffalo, May 21, 22, 23, 1895. Dr. Albert H. Briggs, Major and Surgeon N. G. S. N. Y., 267 Hudson street, Buffalo, N. Y., is chairman of the committee of arrangements. The following are

SOME OF THE SUBJECTS FOR DISCUSSION:

(1) The president's address, by Brigadier-General George M. Sternberg, Surg.-Gen., U. S. Army. (2) Report on the status of the National Guard, by Col. Nelson H. Henry, Asst. Surgeon-General, N. G. S. N. Y. (3) Experiments illustrating the degree of powder burn as modified by the distance of the object, size and conformation of the bore, amount and standard of the powder and other practical demonstrable causes, by Louis A. La Garde, Captain and Assistant Surgeon, U. S. Army. (4) The location and removal of missiles from the cranial cavity, by George R. Fowler, Major and Surgeon, N. G. S. N. Y. (5) Discussion of the report of the committee on litter, by John Van R. Hoff, Major and Surgeon, U. S. Army, and E. T. T. Marsh, Major and Surgeon, 71st Regt., N. G. S. N. Y. (6) Ambulance construction, by Dallas Bache, Lieut.-Col., Deputy Surgeon-General, U. S. Army, and Charles R. Greenleaf, Lieut.-Col. Deputy Surgeon-General, U. S. Army. (7) Conservative surgery on the battlefield, by Nicholas Senn, Surgeon-General, N. G. S. Illinois. (8) The relation of concentrated food to active service demands, by Austin Flint, M. D., ex-Surgeon-General, N. G. S. N. Y. (9) The situation of sites for and the construction of military posts in relation to proper sanitation, by Dallas Bache, Lieut.-Col. Deputy Surgeon-General, U. S. Army. (10) The relation of naval architecture to proper sanitation, by J. R. Tryon, Surgeon-General, U. S. Navy. (11) On the value of bromine in military surgery, by M. O. Terry, Surgeon-General, State of New York. (12) The handling and care of wounded on shipboard, by A. C. H. Russell, Passed Assistant Surgeon, U. S. Navy; E. R. Stitt, Passed Assistant Surgeon, U. S. Navy, and A. M. D. McCormick, Passed Assistant Surgeon, U. S. Navy. (13) The post exchange from a medical standpoint, by Philip F. Harvey, Major and Surgeon, U. S. Army. (14) Infected bullets, by Louis A. La Garde, Captain and Assistant Surgeon, U. S. Army. (15) Instruction of the hospital corps, by H. S. Turrill, Major and Surgeon, U. S. Army. (16) Fifty-two amputations of the thigh, by John D. McGill, Surgeon-General, N. G. S. N. J. (17) Upon the Surgeons-General of the militia, by George Cook, Ex-Surgeon-General, N. G. S. N. H. (18) Field hospital service, by Dallas Bache, Lieutenant-Colonel Deputy Surgeon-General, U. S. Army. (19) Some experimental work with the new ball, by J. D. Griffith, Ex-Surgeon-General, N. G. S. Mo. (20) Gunshot wound of the Kidneys, by Lieutenant-Colonel A. L. Wright, N. G. S. Iowa. (21) On the Travois litter, by Wladimir F. de Niedman, Major and Surgeon, N. G. S. Kans. (22) Details regarding the medical service of the National Guard of the State of New York during the Buffalo strike of 1892, by Louis Balch, Major and Surgeon, N. G. S. N. Y. (23) Details regarding the Medical Service of the National Guard of the State of New York during the recent Brooklyn strike, by Wm. E. Spencer, Major and Surgeon, N. G. S. N. Y. (24) Details regarding the medical service of the National Guard of the State of Illinois during the recent Chicago strike, by Charles Adams, Major and Surgeon, N. G. S. Ills. (25) Method of caring for wounded in field and hospital of Chinese and Japanese armies, by C. U. Gravatt, Surgeon, U. S. Navy. (26.) A consideration of scorbutic manifestations in young subjects, by John C. Wise, Surgeon, U. S. Navy. (27) Asepsis in military service, by Eduard Boeckmann, Asst. Surg.-General, N. G. S. Minn. (28) A medical officer in the volunteer army, by F. W. Byers, Surgeon-General, N. G. S. Wis. (29) The Merriam Pack, by Dr. LewisBalch, Major and Surgeon, N. G. S. N. Y. (30) Measures for the prevention and suppression of dangerous contagious diseases in garrison and in the field, by

H. Lincoln Chase, Lieut. and Asst. Surgeon, Fifth Inf., Mass. Vol. Militia. (31) The effects and treatment of heat and sun-strokes at camps of instruction, by Orlando J. Brown, Lieut. and Asst. Surgeon, Second Inf., Mass. Vol. Militia. (32) The mental evolution of the citizen soldier, by Charles W. Calloupe, Asst. Surgeon, Battery A, Mass. Vol. Militia.

---

THE American Medical Publishers' Association will hold its next annual meeting at Baltimore, May 6, 1895, convening in the parlors of the Eutaw House at 9.30 A. M. Dr. Landon B. Edwards, editor and publisher of the *Virginia Medical Monthly*, is president of the association, and under his direction an interesting program is preparing. All who wish to read papers should furnish the titles to Charles Wood Fassett, secretary, St. Joseph, Mo., at an early day.

---

THE Tri-State medical society of Iowa, Illinois and Missouri will hold its next meeting in St. Louis, April 2, 3, 4, 1895, under the presidency of Dr. James Moores Ball, of that city. The headquarters of the society will be at the Planters' hotel and the sessions will be held in the ladies' ordinary. An elaborate program has been prepared, a reduction of hotel and railway rates secured and a banquet will be given on the evening of April 3d.

